# Engineering a Novel AgMn_2_O_4_@Na_0.55_Mn_2_O_4_ Nanosheet toward High-Performance Electrochemical Capacitors

**DOI:** 10.3390/nano12091538

**Published:** 2022-05-02

**Authors:** Guiling Wang, Zihao Liu, Chenchao Ma, Zhiling Du, Dongyan Liu, Kun Cheng, Xiangju Ye, Tingting Liu, Lei Bai

**Affiliations:** 1 College of Chemistry and Materials Engineering, Anhui Science and Technology University, Bengbu 233030, China; wangguilingcg@126.com (G.W.); lzh2304971627@163.com (Z.L.); mcc13212022@163.com (C.M.); eivleivl@163.com (D.L.); ck482845261@163.com (K.C.); yexiangju555@126.com (X.Y.); 2 School of Energy and Environmental, Hebei University of Engineering, Handan 056038, China; 3 Provincial Key Laboratory of Polyolefin New Materials, College of Chemistry & Chemical Engineering, Northeast Petroleum University, Daqing 163318, China; 2008little@163.com; 4 Northeast Petroleum University at Qinhuangdao, Qinhuangdao 066004, China

**Keywords:** supercapacitor, two-dimensional (2D) materials, Na_0.55_Mn_2_O_4_, AgMn_2_O_4_

## Abstract

Manganese oxides, as a type of two-dimensional (2D) material with high specific area and low cost, are considered promising energy storage materials. Here, we report novel AgMn_2_O_4_/Na_0.55_Mn_2_O_4_ nanosheets created by a popular liquid precipitation method with different AgNO_3_ contents, and their corresponding physical and electrochemical characterizations are performed. The results show that the ultra-thin Na_0.55_Mn_2_O_4_ nanosheets were combined with the AgMn_2_O_4_ nanoparticles and an enhancement in their specific capacity was observed compared to the pristine sheets. This electrode material displays a peak specific capacitance of 335.94 F g^−1^ at 1 A g^−1^. Using an asymmetric supercapacitor (ASC) assembled using a positive electrode made of AgMn_2_O_4_/Na_0.55_Mn_2_O_4_ nanosheets and a reduced graphene oxide (rGO) negative electrode, a high energy density of 65.5 Wh kg^−1^ was achieved for a power density of 775 W kg^−1^. The ASC showed good cycling stability with a capacitance value maintained at 90.2% after 10,000 charge/discharge cycles. The excellent electrochemical performance of the device was ascribed to the heterostructures and the open space formed by the interconnected manganese oxide nanosheets, which resulted in a rapid and reversible faraday reaction in the interface and further enhanced its electrochemical kinetics.

## 1. Introduction

Supercapacitors are considered to be some of the most promising candidates among energy-storage devices due to their inherent advantages, such as high specific capacitance, high power density, superior rate capability, outstanding durability, and excellent environmental compatibility [1,2,3]. The electrode materials of supercapacitors play a crucial role in energy storage through electrical double layer capacitors (EDLC) and pseudocapacitors. In recent years, various electrode materials have been reported in the field of supercapacitors, such as carbon materials [4,5,6], transition metal oxides, [7,8,9,10,11], sulfide [12,13], polyaniline [8,14], and metal organic frameworks [15,16,17].

Two-dimensional (2D) manganese oxides, as hot energy storage materials for batteries, have attracted extensive attention due to their unique physical and chemical properties since they were discovered [18,19,20,21]. Currently, 2D materials are widely selected as electrode materials to enhance specific capacitance due to their high specific surface area for double-layer capacitance and more active sites for pseudocapacitance. However, their characteristics of reunion and volume expansion/contraction during the charge-discharge process greatly limit their advantages, resulting in descending cyclic stability. Thus, many efforts have also been made to improve electrochemical performance by coupling the active phase with conductive carbon materials and doping heteroatom [22,23]. Recently, Wan et al. [24] found that δ-Al_0.06_MnO_2_ showed a larger specific capacitance of 450 mF cm^−2^ at a current density of 0.1 mA cm^−2^ than that of δ-MnO_2_ (420 mF cm^−2^ at 0.1 mA cm^−2^) and that its capacitance retention was enhanced from 75% for pure δ-MnO_2_ to 84% in δ-Al_0.1_MnO_2_ after 5000 cycles. Hu et al. [25] prepared Cu^2+^ intercalated δ-MnO_2_ and, through bader charge analysis, demonstrated that the Cu^2+^ intercalation decreased the average valence state of Mn, thus creating more redox-active sites in bulk MnO_2_. Zarshad et al. [26] reported on the core-shell heterostructure of birnessite-type Fe-doped manganese oxides. The Fe-MnO_2_ electrodes displayed a specific capacitance of about 350 F g^−1^ at a current density of 1 A g^−1^ and presented an excellent cycling performance with capacitance retention of 88.8% after 6000 cycles at a current density of 10 A g^−1^. The above studies confirm that heteroatom doping is considered an effective method for improving the properties of manganese dioxide electrode materials.

In this paper, novel AgMn_2_O_4_@Na_0.55_Mn_2_O_4_ nanosheets were prepared by liquid phase precipitation method. Silver ions were first precipitated into Ag_2_O nanoparticles, subsequently converted into AgMn_2_O_4_ and then further intercalated onto the growing Na_0.55_Mn_2_O_4_ nanosheets, which significantly enhances the electron transferability between the conductive AgMn_2_O_4_ particles and Na_0.55_Mn_2_O_4_ nanosheets. In addition, the influence of morphology on the electrochemical properties of the doped products was studied. AgMn_2_O_4_@Na_0.55_Mn_2_O_4_ displayed the best specific capacitance of 335.94 F g^−1^ at 1 A g^−1^, which was higher than the Na_0.55_Mn_2_O_4_ nanosheets’ capacitance of 118.7 F g^−1^.

## 2. Materials and Methods

### 2.1. Material Synthesis

All reagents were of analytical grade and were used as received without further purification. AgMn_2_O_4_@Na_0.55_Mn_2_O_4_ nanosheets were synthesized using a facile chemical deposition method. Typically, 0.0028 mol manganese acetate and a certain silver nitrate content, 1.16 g EDTA and 50 mg SDS were dissolved in 50 mL of deionized water and stirred for about 1 h. Note that silver nitrate content was controlled by tuning the molar ratio for silver to manganese to 0%, 5%, 10%, and 15%, respectively. Then, 50 mL of 0.25 M NaOH aqueous solution was subsequently added to the above solution and 50 mL 0.12 M K_2_S_2_O_8_ aqueous solution was dripped into the mixture solution and stirred continuously to obtain the precipitation product through a chemical deposition reaction. Finally, the solution was maintained at a constant temperature in a water bath of 30 °C for 12 h, resulting in the targeted production of silver-doped ultrathin manganese oxide nanosheets. The samples synthesized with different silver to manganese molar ratios of 0%, 5%, 10%, 15% were respectively notated as Mn-Ag-0.00, Mn-Ag-0.05, Mn-Ag-0.10, and Mn-Ag-0.15.

### 2.2. Structure Characterization

The powder X-ray diffraction (XRD) patterns were carried out on a RigakuSmart Lab X-ray diffractometer (Jena, Germany) with Cu Kα radiation (λ = 0.1541 nm, operated at 40 kV and 40 mA) at a scanning rate of 20° min^−1^ to determine the structures of samples. Transmission electron microscopy (TEM) was performed on a Hitachi HT7700 (Tokyo, Japan) at an acceleration voltage of 120 kV and scanning electron microscope (SEM) images were gained using a Carl Zeiss Super55 (Jena, Germany) operating at an accelerating voltage of 5 kV to examine the morphologies and microstructures. X-ray photoelectron spectroscopy (XPS) studies were conducted on a Thermo (Shanghai, China) using Al K (=1486.6 eV) radiation, operated at an accelerating voltage of 12.5 kV.

### 2.3. Electrochemical Properties

All related electrochemical characterizations were measured by a CHI 660E electrochemical workstation (CH Instrument, Chenhua Co., Shanghai, China) in 6 M KOH via a typical three-electrode system. The preparation of MnO_2_-based electrodes and electrochemical characterizations were similar to our previously reported work [27], and the loading amount of the active material was about 3.0 mg cm^−2^ in electrochemical experiments. A two-electrode system was designed to analyze the power and energy density of a manganese oxide asymmetric supercapacitor (Mn-Ag-0.10//rGO). A battery test instrument system (BST4008, Xinwei, China) was used to characterize the cycling performance of the ASC device. The ASC devices were prepared using nanomaterials (Mn-Ag-0.10) as the positive electrode with the rGO as the negative electrode (Figures S1–S3) in 6 M KOH electrolyte. The mass ratio between the positive and negative electrode materials was 1:2. The stable potential window was −1.0~−0.2 V for the rGO electrode and 0~0.55 V for the Ag-0.10 electrode. The energy density was calculated by using the following equation:

$$E=1/2C_{\mathrm{cell}}V^{2}$$

where C_cell_ is the total cell specific capacitance and V is the cell-operation potential. The average power density was calculated by using the following equation:

P=E/Δt

where E is the energy density and Δt is the discharge time.

## 3. Results and Discussion

**Figure 1:**
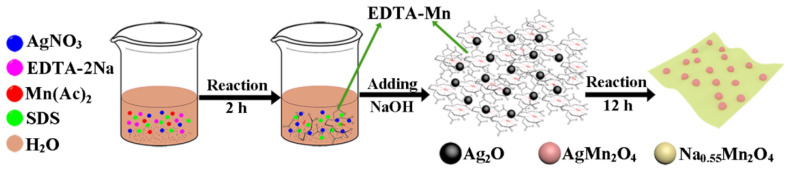
Schematic illustration of the formation mechanism of AgMn_2_O_4_@Na_0.55_Mn_2_O_4_ nanosheets.

The formation mechanism of AgMn_2_O_4_@Na_0.55_Mn_2_O_4_ nanolamellar structure is shown in Figure 1. When EDTA-Na_2_ was added to an Mn(Ac)_2_-AgNO_3_ mixed solution, it was easier to generate a stable EDTA-Mn complex due to the relatively large coordination constant K_f_ (lgK_f_ = 13.87) between Mn^2+^ and EDTA. However, the coordination constant K_f_ of EDTA-Ag formed by Ag^+^ and EDTA was relatively small (lgK_f_ = 7.32). When the precipitant NaOH was added, free Ag^+^ would first precipitate with NaOH to generate AgOH and further dehydrate to form black Ag_2_O nanoparticles. Conversely, the precipitation process generated by Mn^2+^ and NaOH was slow for a large coordination equilibrium constant K_f_. AgMn_2_O_4_ was formed and intercalated onto the growing Na_0.55_Mn_2_O_4_ nanosheet by the reciprocal actions between Ag_2_O nanoparticles and EDTA-Mn. Owing to the in-situ growing process, the AgMn_2_O_4_ and Na_0.55_Mn_2_O_4_ interacted with each other by intermolecular forces. The formation of a heterojunction structure could lead to excellent structural stability, which would allow the electrode to maintain good electrochemical performance during the charge-discharge process. 

### 3.1. XRD Analysis

**Figure 2:**
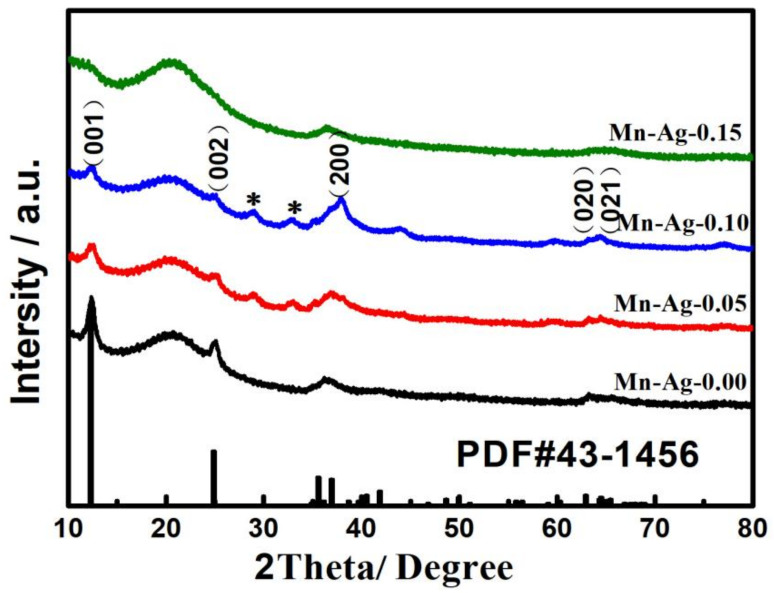
XRD patterns of Mn-Ag-0.00, Mn-Ag-0.05, Mn-Ag-0.10, and Mn-Ag-0.15 samples.

It is noted from Figure 2 that the XRD patterns of Mn-Ag-0.00, Mn-Ag-0.05, Mn-Ag-0.10, and Mn-Ag-0.15 show obvious diffraction peaks, attributed to the Na_0.55_Mn_2_O_4_ (PDF#43-1456). The XRD patterns of Mn-Ag-0.05 and Mn-Ag-0.10 present the minor peaks of the impurities indicated by the asterisk, which were indexed to AgMn_2_O_4_(PDF#16-0740). However, the diffraction peaks for Na_0.55_Mn_2_O_4_ and AgMn_2_O_4_ weakened and no AgMn_2_O_4_-related peaks were detected as the molar ratio of silver to manganese increased to 15% (Mn-Ag-0.15). From this phenomenon, it is speculated that silver had a great effect on the crystallinity of Na_0.55_Mn_2_O_4_ by affecting the ion as a liquid, and the Na_0.55_Mn_2_O_4_ directly related to the formation of AgMn_2_O_4_.

**Figure 3:**
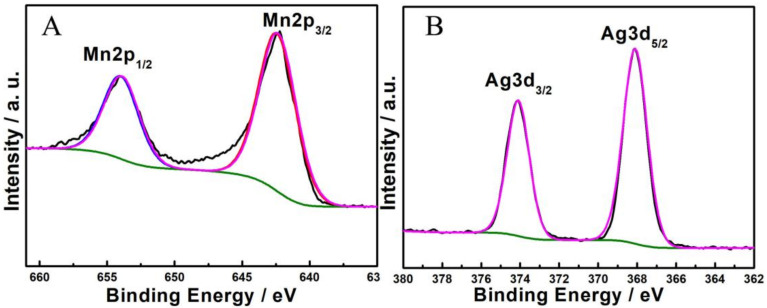
XPS of Mn-Ag-0.10: (**A**) Mn 2p (**B**) Ag 3d.

XPS measurements were performed to investigate the surface metal oxidation states of Mn-Ag-0.10. Figure 3A shows the M 2p transitions. The binding energies of Mn 2p_3/2_ and Mn 2p_1/2_ are 642.33 and 653.9 eV, which can be attributed to a mixture of Mn^4+^ and Mn^3+^ [28]. Figure 3B displays the XPS of Ag 3d. The Ag 3d_5/2_ and Ag 3d_3/2_ peaks are 368.1 and 374.1 eV, which are characteristic of Ag^+^ [29].

### 3.2. Effect of Doping on Morphology

**Figure 4:**
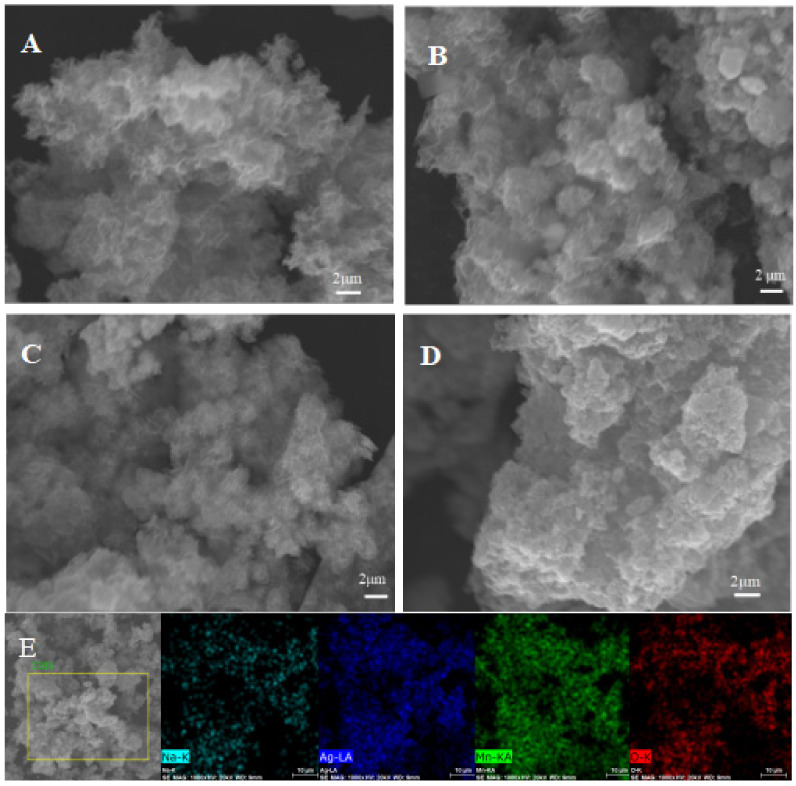
SEM images of (**A**) Mn-Ag-0.00, (**B**) Mn-Ag-0.05, (**C**) Mn-Ag-0.10, (**D**) Mn-Ag-0.15, and mapping of (**E**) Mn-Ag-0.10.

The SEM images of Ag-0.00, Ag-0.05, Ag-0.10, and Ag-0.15 are shown in Figure 4. It was suggested that the morphological qualities of the materials changed obviously after adding silver ions. The material presented an irregular layered structure (Figure 4A) when no silver was added. The lamellars recombined into spherical nanoflowers after the silver with the the molar ratio to manganese of 5% and 10% were added, as shown in Figure 4B,C. The lamellar and spherical nanoflower structures provided high specific surface area and many pores, which could be conduced to expose more active area for charge storage, shorten the charge transfer pathway, and accelerate the electrochemical reaction, respectively. However, with an increase of the silver ion concentration to 15% (Figure 4D), the solid was agglomerated into particles. EDS (Figure 4E and Figure S4) of Ag-0.10 was used to analyze the distribution of silver in the sample and it was found that the silver was uniformly distributed and no large area partial set was noticed.

**Figure 5:**
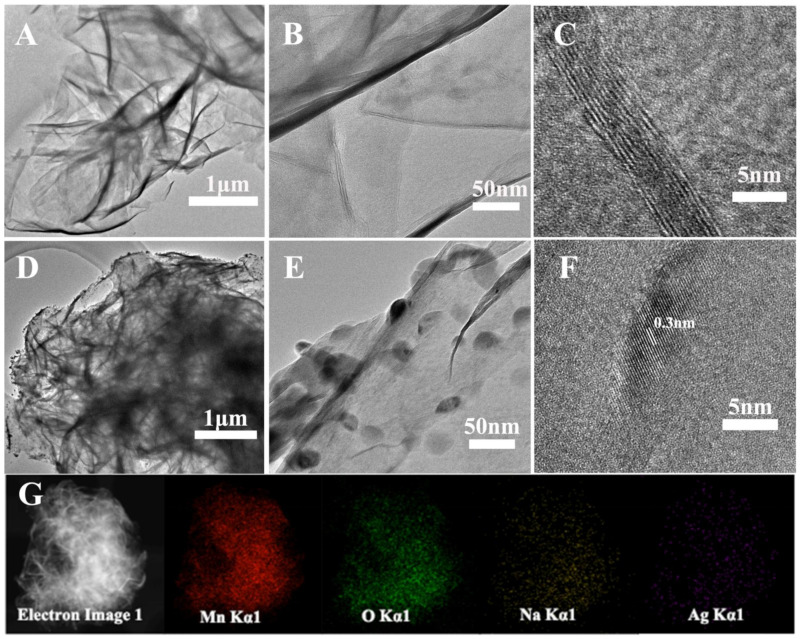
TEM images of Mn-Ag-0.00 (**A**–**C**), Mn- Ag-0.10 (**D**–**F**), and mapping of Mn-Ag-0.10 (**G**).

In addition, transmission electron microscopy (TEM) was performed on Mn-Ag-0.00 and Mn-Ag-0.10 and their morphological and internal structure properties were analyzed. In Figure 5A–C, Mn-Ag-00 shows an ultra-thin nanosheet structure. This nanosheet was a multi-layer structure formed by about ten layers of manganese oxide. After Ag^+^ was added (Figure 5D–F), the finer flaky spherical aggregates formed and many small particles adhered to the surface of the nanosheet. A HRTEM image (Figure 5F) displays that the small particles had lattice fringes of 0.3 nm and could be identified as AgMn_2_O_4,_ while the manganese oxide had no obvious lattice structure. Figure 5G shows the EDS mapping of Figure 5D. It can be seen that AgMn_2_O_4_ was uniformly dispersed on the Na_0.55_Mn_2_O_4_ nanosheets, which is consistent with the results of SEM. The homogeneous distribution of Ag and Mn elements illustrated the in situ formation of AgMn_2_O_4_ in the Na_0.55_Mn_2_O_4_ matrix, a state which will typically result in the formation of heterostructures and intimate contact between the components [30,31]. 

**Figure 6:**
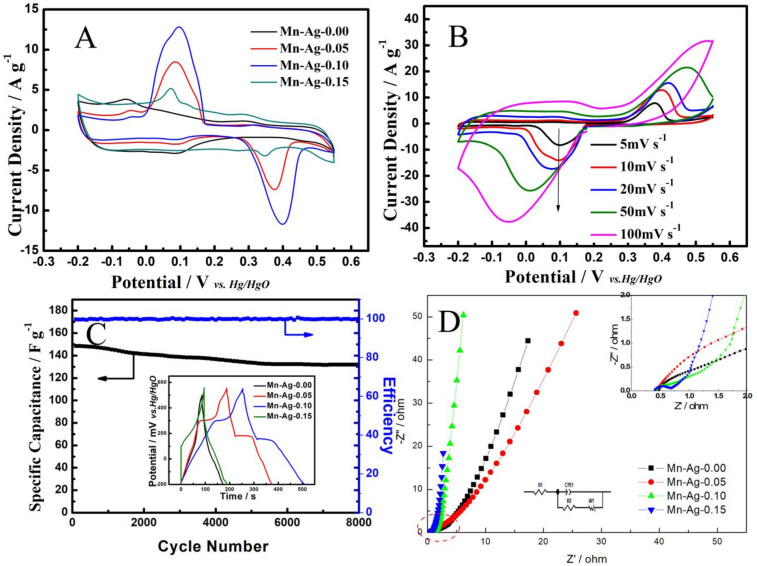
Electrochemical performance of synthesized samples. (**A**) CV curves at the scan rate of 10 mV s^−1^. (**B**) CV curves of Ag-Mn-0.10 at different scan rates. (**C**) Lifetime and coulombic efficiency curves of Ag-Mn-0.10 at a high current density of 10 A g^−1^. The insert in the bottom-left corner is the galvanostatic charge-discharge curves tested at 1 A g^−1^. (**D**) Nyquist plots. The insert in the top-right corner is the enlarged Nyquist plot in the high frequency region.

To investigate the capacitive property of the new phase of AgMn_2_O_4_@Na_0.55_Mn_2_O_4_, we first evaluated the CV curves of the different samples, as shown in Figure 6A. The curves suggest a pair of distinct redox peaks at ca. 0.4 V and 0.1 V, which resulted from the insertion/extraction of ions during the charge and discharge process [32], the increased trend of current intensity with the added silver content, and the decreased trend as the molar ratio rose up to 15%. It is obvious that the maximum current response was achieved when the silver content was 10%. This result indicates that the sample of Mn-Ag-0.10 possessed the best pseudocapacitance properties, ascribed to the formation of a new phase of AgMn_2_O_4_. The values of these samples were 167.84 F g^−1^, 233.54 F g^−1^, 248.23 F g^−1^, and 191.92 F g^−1^. 

Figure 6B shows the CV curves of the Mn-Ag-0.10 sample at various scanning rates (Mn-Ag-0.00, Mn-Ag-0.05, and Mn-Ag-0.15 are shown in Figure S5). The consecutive order change of the shapes of the CV curves with a scanning rate from 5 to 100 mV s^−1^ indicates reasonable capacitive behaviors and high-rate cyclability and stability. It can be calculated that the specific capacitances of Mn-Ag-0.10 at scanning rates of 5, 10, 20, 50, and 100 mV s^−1^ are 315.17, 248.23, 151.82, 139.51, and 101.19 F g^−1^, respectively.

The galvanostatic charge-discharge curves are described in the insert of Figure 6C, where the electrodes were tested at 1 A g^−1^. The values of Mn-Ag-0.00, Mn-Ag-0.05, Mn-Ag-0.10 and Mn-Ag-0.15 were 117.36 F g^−1^, 250.15 F g^−1^, 335.94 F g^−1^, and 132.02 F g^−1^. This change trend is consistent with the CV images. It can be seen that the Mn-Ag-0.10 (GCD curves of different samples tested at 2 A g^−1^, 5 A g^−1^, and 10 A g^−1^ are shown in Figure S6 and Table S1) exhibited the highest specific capacitance, which is consistent with the CV curves. Cycle stability is an important parameter in industrial fields and is explored in detail in Figure 6C. The Mn-Ag-0.10 sample at a high current density of 20 A g^−1^ presented excellent cycle durability with a high capacity retention of 88.80% from 148.46 F g^−1^ to 131.83 F g^−1^ after 8000 cycles. Additionally, the coulombic efficiency of Mn-Ag-0.10 was near 100%, which was due to the rapid and reversible faraday reaction in the interface between the active material and electrolyte.

To further explore the capacitive behaviors of the prepared samples, EIS analysis was performed over a frequency range from 100 kHz to 10 mHz. As clearly displayed in Figure 6D, the Nyquist plots in the low-frequency region are steeply and more perpendicular to the real axis with the addition of the Ag-doped content. The plots of Ag-0.10 and Ag-0.15 display almost vertical slopes, indicating efficient diffusion of electrolyte ions to active material surfaces. Moreover, the Nyquist plots of all samples demonstrate shot x-intercepts and small diameters of the semicircle in high- and mild-frequency regions (Table S2). Mn-Ag-0.15 presents the smallest diameter, owing to the formation of the new phase, AgMn_2_O_4_. In view of all the above, the introduction of AgMn_2_O_4_ can lead to small charge-transfer resistances and superior capacitive performance. However, micromorphology plays a critical role in charge storage for supercapacitors, as suggested by the fact that the Mn-Ag-0.10 electrode exhibited the best electrochemical properties of all electrodes considering the combination effect of electrical conductivity and micromorphology.

The construction of an ASC device is generally considered an effective strategy to evaluate the application value of as-prepared electrodes in a real system [33,34,35,36].

**Figure 7:**
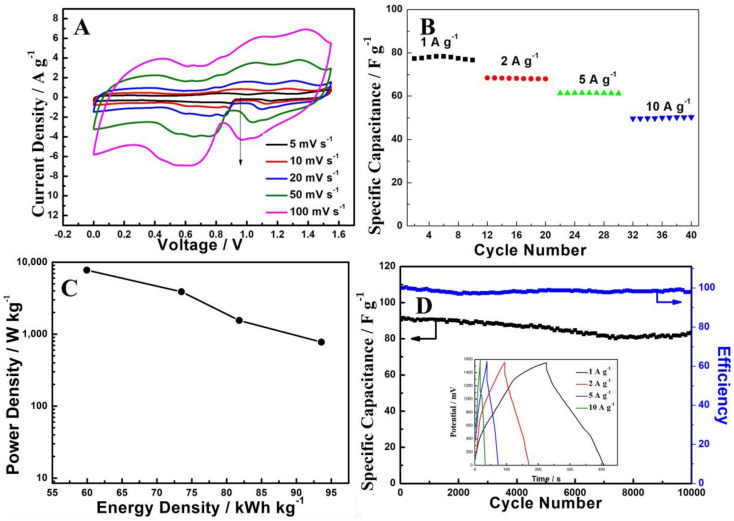
(**A**) CV curves of Mn-Ag-0.10//rGO at various scan rates (5–100 mV s^−1^); (**B**) Rate performance of Ag-0.10//rGO; (**C**) Ragone plot of the energy density and the power density of Mn-Ag-0.10//rGO; (**D**) Cycling stability and coulombic efficiency of Mn-Ag-0.10//rGO. The insert is the galvanostatic charge-discharge curves tested at 1 A g^−1^.

The ASC of Mn-Ag-0.10//rGO at different scanning rates (5–100 mV s^−1^) are shown in Figure 7A. A symmetrical CV shape was still maintained well even at scanning rates up to 100 mV s^−1^, suggesting that the ASC device had a desirable charge-discharge property and perfect supercapacitor behavior. The rate performances for ASC device are shown in Figure 7B. The Mn-Ag-0.10//rGO was 78.3 F g^−1^ at 1 A g^−1^, and when the current density rose up to 10 A g^−1^, the capacitance value still remained at 49.9 F g^−1^. The Ragone plot of the power density and the energy density of an asymmetric supercapacitor are crucial for evaluating the effect of a capacitor (Figure 7C). The Mn-Ag-0.10//rGO supercapacitor achieved an energy density of 65.5 kWh kg^−1^ at a power density of 775 W kg^−1^. This supercapacitor maintained an energy density of 41.98 kW h kg-1 with a power density up to 7750 W kg^−1^. The cycling stability and efficiency of this supercapacitor was measured by GCD test (Figure 7D, the inset of Figure 7D are GCD curves at different densities after 10,000 cycles). After 10,000 charge−discharge cycles at 1 A g^−1^, the Mn-Ag-0.10//rGO electrode still maintained 90.2% capacitance retention. The electrochemical properties of the nanosheets of manganese oxides and their composite materials reported in the literature are listed in Table 1. It can be seen from the literature comparision that the Mn-Ag-0.10//rGO ASC prepared in this work is comparable. Its excellent electrochemical performance could be ascribed to the following advantages. On the one hand, the open space formed by the interconnected manganese oxide nanosheets greatly facilitates the transport of electrolyte, thus enhancing electrochemical kinetics. On the other hand, the heterostructures have excellent structural stability, which ensures the electrode maintains good electrochemical performance during the charge-discharge process.

## 4. Conclusions

In summary, novel AgMn_2_O_4_/Na_0.55_Mn_2_O_4_ nanosheets were synthesized with a popular liquid precipitation method for use as high-performance electrodes for supercapacitors. TEM images show that AgMn_2_O_4_ formed in situ in the Na_0.55_Mn_2_O_4_ matrix, which resulted in the formation of heterostructures and the intimate contact between the components. The combination of AgMn_2_O_4_ nanoparticles and ultra-thin Na_0.55_Mn_2_O_4_ nanosheets provided the electrodes with heterostructures with fast ion transport and a synergistic effect from the AgMn_2_O_4_/Na_0.55_Mn_2_O_4_ hybrid electrodes. This electrode material displayed a peak specific capacitance of 335.94 F g^−1^ at 1 A g^−1^ in a three-electrode system. In addition, its discharge capacity at 10 A g^−1^ was slightly decreased, after 8000 cycles, fading from 148.46 to 131.83 F g^−1^, and the corresponding capacitance retention was 88.8%. The Mn-Ag-0.10//rGO ASC had a high energy density of 65.5 Wh kg^−1^ at a power density of 775 W kg^−1^ and showed good cycling stability, with 90.2% capacitance value maintained after 10,000 charge/discharge cycles.

## Figures and Tables

**Table 1:** Comparison of the electrochemical performance of AgMn_2_O_4_/Na_0.55_Mn_2_O_4_ nanosheets with other previously reported two-dimensional manganese oxides.

Materials	Specific Capacitance	Cycle Stability	Power Density	Energy Density	Reference
C@MnO nanosheets	162.7 F g^−1^(0.5 A g^−1^)	93.5% (10 A g^−1^) 5000 cycles (ASC)	400 W kg^−1^	57.7 Wh kg^−1^	[19]
MnCo_2_O_4_ nanoflakes	256 F g^−1^(5 mV s^−1^)	85% (2 A g^−1^) 10,000 cycles (ASC)	1000 W kg^−1^	25 Wh kg^−1^	[37]
lamellarMnO_2_@Carbon nanocoil	435 F g^−1^(1 A g^−1^)	92.7% (2 A g^−1^) 5000 cycles (ASC)	100 W kg^−1^	21.58 Wh kg^−1^	[38]
CoMn_2_O_4_nanosheets /carbon nanotubes	732 F g^−1^(2 mV s^−1^)	77% (100 mV s^−1^) 5000 cycles (ASC)	400 W kg^−1^	47.39 Wh kg^−1^	[39]
Fe doped MnO_2_ nanosheets	157 F g^−1^(0.5 A g^−1^)	71.4% (0.5 A g^−1^) 5000 cycles	1000 W kg^−1^	30.3 Wh kg^−1^	[40]
AgMn_2_O_4_/ Na_0.55_Mn_2_O_4_ nanosheets	335.94 F g^−1^(1 A g^−1^)	90.4% (10 A g^−1^) 10,000 cycles (ASC)	775 W kg^−1^	65.5 Wh kg^−1^	This work

## Data Availability

The data presented in this study are available on request from the corresponding author.

## Supplementary Materials

The following supporting information can be downloaded at: https://www.mdpi.com/article/10.3390/nano12091538/s1, Figure S1: CV curves of rGO; Figure S2: The galvanostatic charge-discharge curve of rGO tested at 1 A g^−1^; Figure S3: Cycle life curve of rGO at a current density of 2 A g^−1^; Figure S4. EDS of Mn-Ag-0.10; Figure S5: CV curves of Mn-Ag-0.00,Mn-Ag-0.05, Mn-Ag-0.15 at different scan rates; Figure S6: GCD curves of Mn-Ag-0.00,Mn-Ag-0.05, Mn-Ag-0.10 and Mn-Ag-0.15 at (A) 2 A g^−1^, (B) 5 A g^−1^ and (C) 10 A g^−1^; Table S1: Specific Capacitance of Mn-Ag-0.00, Mn-Ag-0.05, Mn-Ag-0.10 and Mn-Ag-0.15 at different current density; Table S2: Rs and Rct values of Mn-Ag-0.00, Mn-Ag-0.05, Mn-Ag-0.10 and Mn-Ag-0.15 samples.
