# Supplementary material for: Engineering a Novel AgMn2O4@Na0.55Mn2O4 Nanosheet toward High-Performance Electrochemical Capacitors
Source: Nanomaterials (Basel). 2022 May 2;12(9):1538. doi: 10.3390/nano12091538 (PMC9104129; doi:10.3390/nano12091538)
Supplement: Supplementary file 1 [file nanomaterials-12-01538-s001.zip › nanomaterials-1689682-supplementary.pdf]

*Article*

# Engineering a Novel $AgMn_2O_4@Na_{0.55}Mn_2O_4$ Nanosheet toward High-Performance Electrochemical Capacitors

**Guiling Wang [1], Zihao Liu [1], Chenchao Ma [1], Zhiling Du [1,2,*], Dongyan Liu [1], Kun Cheng [1], Xiangju Ye [1], Tingting Liu [3,4] and Lei Bai [1,*]**

[1] College of Chemistry and Materials Engineering, Anhui Science and Technology University, Bengbu 233030, China; wangguilingcg@126.com (G.W.); lzh2304971627@163.com (Z.L.); mcc13212022@163.com (C.M.); eivleivl@163.com (D.L.); ck482845261@163.com (K.C.); yexiangju555@126.com (X.Y.)
[2] School of Energy and Environmental, Hebei University of Engineering, Handan 056038, China
[3] Provincial Key Laboratory of Polyolefin New Materials, College of Chemistry & Chemical Engineering, Northeast Petroleum University, Daqing 163318, China; 2008little@163.com
[4] Northeast Petroleum University at Qinhuangdao, Qinhuangdao 066004, China
* Correspondence: zhilingdu@hebeu.edu.cn (Z.D.); baileiwj2014@163.com (L.B.)

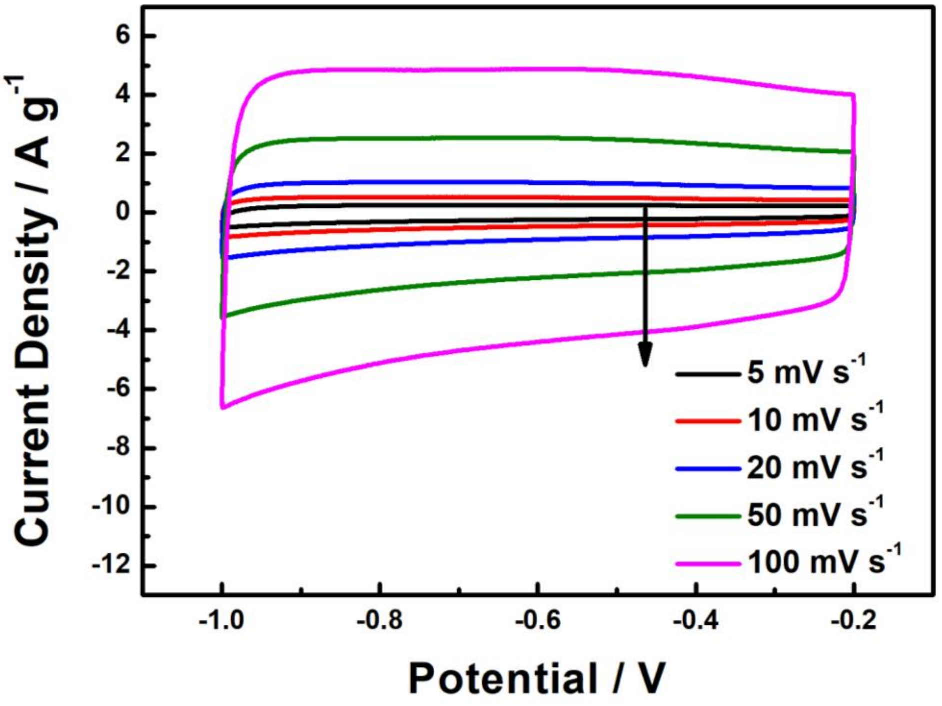


**Figure S1.** CV curves of rGO.

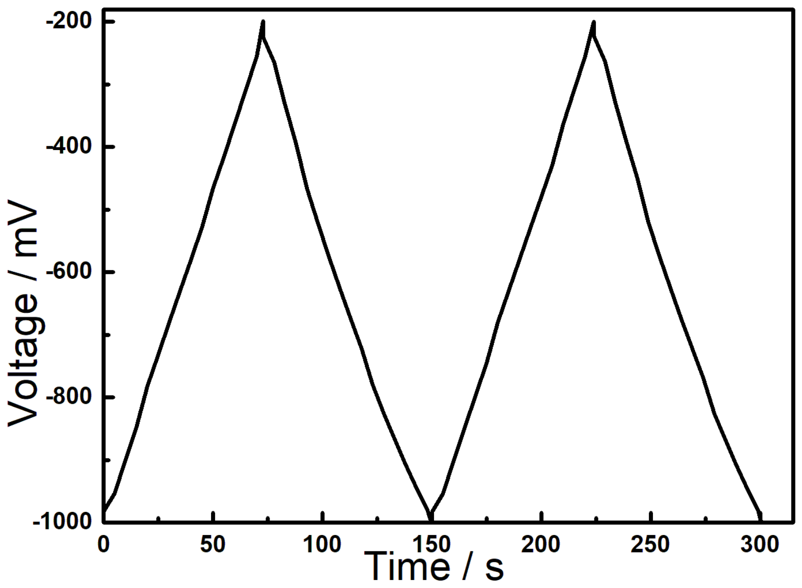


**Figure S2.** The galvanostatic charge-discharge curve of rGO tested at 1 A $g^{-1}$.

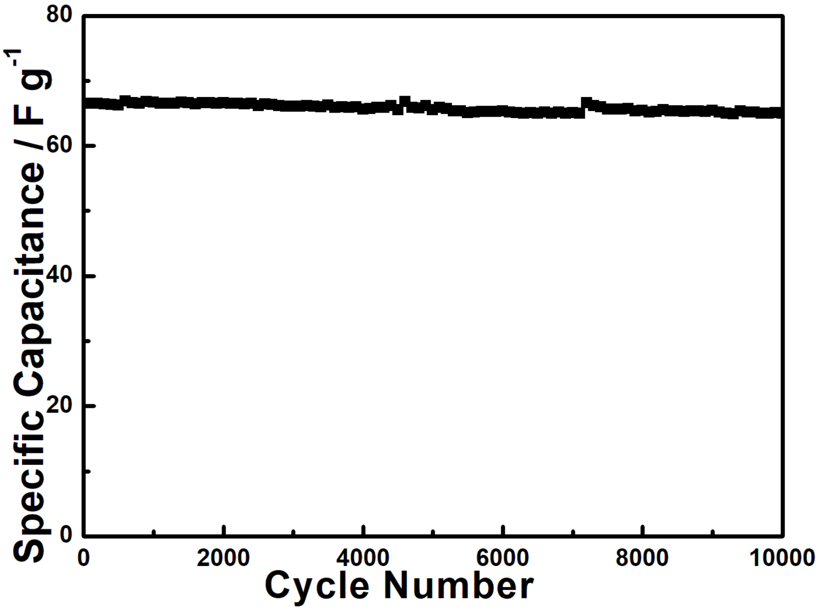


**Figure S3.** Cycle life curve of rGO at a current density of 2 A $g^{-1}$

Spectrum: 3383

| Element | Series | unn. C [wt.%] | norm. C [wt.%] | Atom. C [at.%] | Error (1 Sigma) [wt.%] |
|---|---|---|---|---|---|
| Silver | L-series | 9.86 | 11.06 | 2.45 | 0.68 |
| Oxygen | K-series | 34.78 | 39.01 | 58.24 | 3.86 |
| Manganese | K-series | 32.05 | 35.95 | 15.61 | 0.98 |
| Sodium | K-series | 3.87 | 4.34 | 4.5 | 0.25 |
| Carbon | K-series | 8.59 | 9.64 | 19.19 | 1.61 |

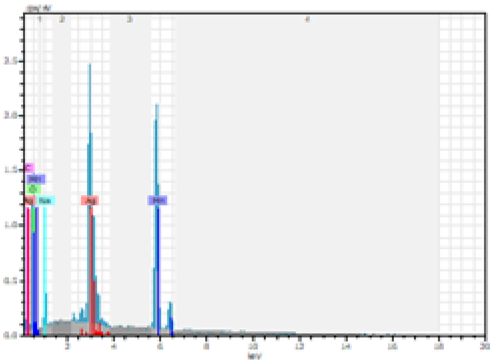

**Figure S4.** EDS of Mn-Ag-0.10.

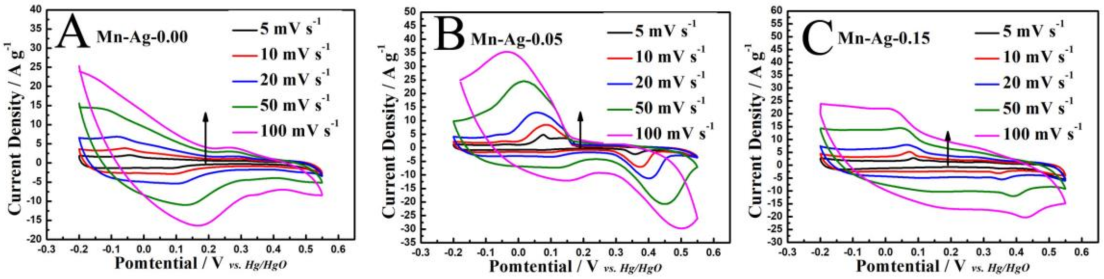


**Figure S5.** CV curves of Mn-Ag-0.00,Mn-Ag-0.05, Mn-Ag-0.15 at different scan rates.

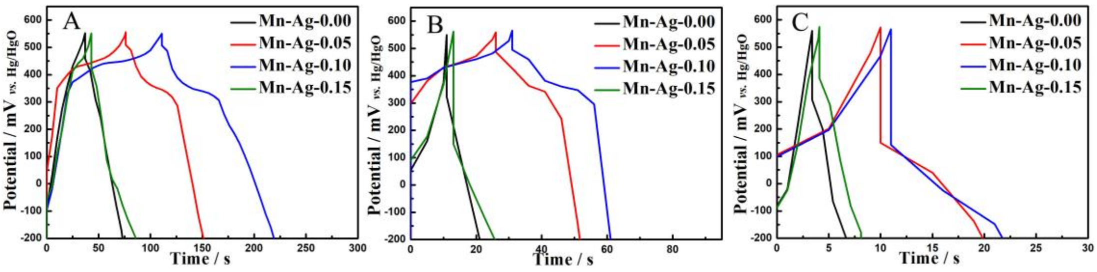


**Figure S6.** GCD curves of Mn-Ag-0.00,Mn-Ag-0.05, Mn-Ag-0.10 and Mn-Ag-0.15 at (**A**) 2 A g$^{-1}$, (**B**) 5 A g$^{-1}$ and (**C**) 10 A g$^{-1}$.

**Table S1.** Specific Capacitance of Mn-Ag-0.00, Mn-Ag-0.05, Mn-Ag-0.10 and Mn-Ag-0.15 at different current density.

| Samples | Mn-Ag-0.00 | Mn-Ag-0.05 | Mn-Ag-0.10 | Mn-Ag-0.15 |
|---|---|---|---|---|
| SC(1 A g$^{-1}$) | 117.36 F g$^{-1}$ | 250.15 F g$^{-1}$ | 335.94 F g$^{-1}$ | 132.02 F g$^{-1}$ |
| SC(2 A g$^{-1}$) | 96.08 F g$^{-1}$ | 202.67 F g$^{-1}$ | 288.32 F g$^{-1}$ | 114.72 F g$^{-1}$ |
| SC(5 A g$^{-1}$) | 66.67 F g$^{-1}$ | 166.75 F g$^{-1}$ | 206.67 F g$^{-1}$ | 82.27 F g$^{-1}$ |
| SC(10 A g$^{-1}$) | 45.34 F g$^{-1}$ | 104.54 F g$^{-1}$ | 157.23 F g$^{-1}$ | 57.16 F g$^{-1}$ |

**Table S2.** Rs and Rct values of Mn-Ag-0.00, Mn-Ag-0.05, Mn-Ag-0.10 and Mn-Ag-0.15 samples.

| Samples | Mn-Ag-0.00 | Mn-Ag-0.05 | Mn-Ag-0.10 | Mn-Ag-0.15 |
|---|---|---|---|---|
| Rs | 0.7476 | 0.43889 | 0.42726 | 0.42099 |
| Rct | 0.47334 | 0.3403 | 0.2878 | 0.2812 |
